# Editorial

**DOI:** 10.4103/0974-9233.53366

**Published:** 2008

**Authors:** Alexander Bialasiewicz

Ophthalmic research is the collection and interpretation of information to explain the function and dysfunction of the eye and to provide practical applications. Clinical as well as basic research is intricately linked to the environment, to society and to the level of development.

At present, ophthalmic research in the Middle East and Africa is not at the level that it should be. The limited resources are vastly channeled towards service delivery. This is true even in countries with adequate financial recourses. Unfortunately human resource development, screening programs and research get less attention compared with immediate service delivery that is sometimes driven by political pressures and the need to achieve quick observable results.

The difficulties encountered in planning preventive and curative programs in the struggle against Visual Impairment (VI) in the region are a prime example of the lack of solid data on the causes and the prevalence of ocular blinding diseases. The two regions with the highest percentage of VI among its population are Africa (7.97%) and the Eastern Mediterranean Region (EMR) (6.54%)^1^ and in spite of that, they are the two most significantly lacking basic, updated and scientifically sound data on prevalence and causes of VI.

In this issue of MEJO several original articles highlight some problems of the region. A significant number of countries in the EMR have not yet conducted and published national eye morbidity and blindness statistics because of lacking of support from local authorities. However, in order to plan healthcare and research for decision-makers, it is essential to know, which diseases should be given priority (Bourne 2007).^2^ The EMR Office of the International Agency for the Prevention of Blindness (IAPB) in collaboration with IMPACT-EMR, a major non-governmental development organization, is lending support to researchers in the EMR by organizing four workshops throughout 2008 on how to conduct a Rapid Assessment for Avoidable Blindness (RAAB)^3^ survey. This simple and rapid survey methodology can provide data on the prevalence and causes of blindness within a province of 0.5 to 5 million people by surveying between 2,000 and 5,000 people. Therefore, it is of great merit that Al-Akily and Bamashmus^4^ have collected data from 3845 patients in a hospital setting. The study has revealed that the most frequent and important causes of blindness are cataracts and trauma representing features of a rural country highlighted in Oman also, and which contrasts against the more urban populations of the Gulf Cooperation Council (GCC) states.

Cultural peculiarities may predispose to increased anxiety during vitreoretinal surgery under local anesthesia, an issue which has been addressed by Abboud, Mansour and Riad^5^ in patients from Saudi Arabia. As has been reported previously from Japan (Sugisaka 2007),^6^ patients are affected negatively by this surgery. Cultural differences obviously do not play a role, which has also been shown in when compared to postoperative anxiety and depression in patients without improvement or deterioration in their vision comparable to a study on cataract patients reported in 2006 (Mitsonis 2006).^7^

Last but not least, the distinct diagnostic features and management of the orbital pseudotumor has been highlighted in the review article of this issue by Chaudhry et all.^8^

Whereas research in genetics and molecular biology are of general interest for a global community and for international journals, well-conceived clinical and epidemiological studies can highlight regional problems, which may be presented by transnational journals. In future, MEJO wishes to provide help and substantive recommendations for regional researchers and programs in order to balance good ideas with appropriate scientific work-up and formal presentation.
